# Supplementary material for: DNA Methyltransferase 1 (DNMT1) Acts on Neurodegeneration by Modulating Proteostasis-Relevant Intracellular Processes
Source: Int J Mol Sci. 2020 Jul 30;21(15):5420. doi: 10.3390/ijms21155420 (PMC7432412; doi:10.3390/ijms21155420)
Supplement: Supplementary file 1 [file ijms-21-05420-s001.zip › Supplementary Figure Legends_final.pdf]

Supplementary Figures and Legends

Figure S1

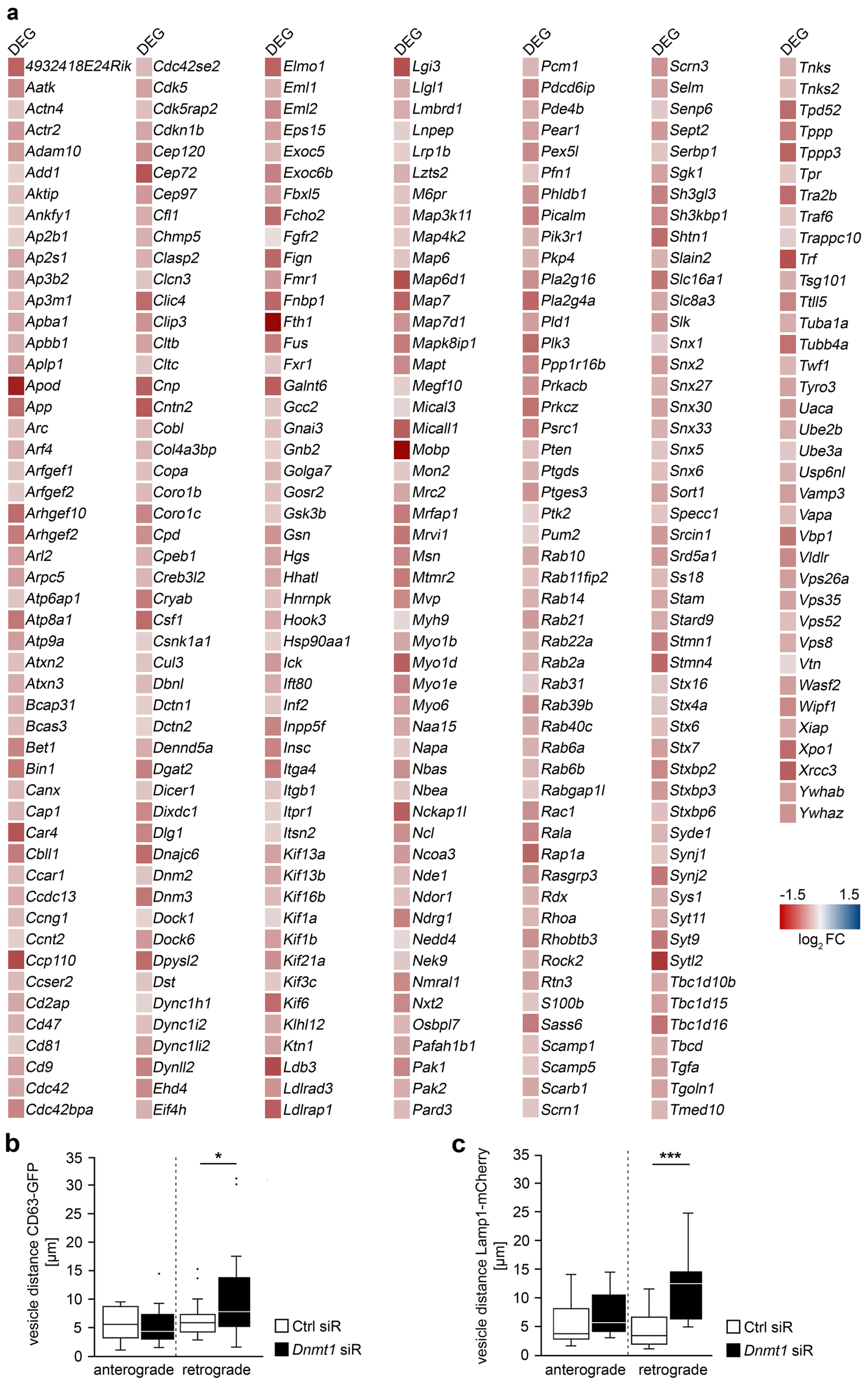
a
DEG
4932418E24Rik
Aatk
Actn4
Actr2
Adam10
Add1
Aktip
Ankfy1
Ap2b1
Ap2s1
Ap3b2
Ap3m1
Apba1
Apbb1
Aplp1
Apod
App
Arc
Arf4
Arfgef1
Arfgef2
Arhgef10
Arhgef2
Arl2
Arpc5
Atp6ap1
Atp8a1
Atp9a
Atxn2
Atxn3
Bcap31
Bcas3
Bet1
Bin1
Canx
Cap1
Car4
Cbll1
Ccar1
Ccdc13
Ccng1
Ccnt2
Ccp110
Ccser2
Cd2ap
Cd47
Cd81
Cd9
Cdc42
Cdc42bpa
DEG
Cdc42se2
Cdk5
Cdk5rap2
Cdkn1b
Cep120
Cep72
Cep97
Cfl1
Chmp5
Clasp2
Clcn3
Clic4
Clip3
Cltb
Cltc
Cnp
Cntn2
Cobl
Col4a3bp
Copa
Coro1b
Coro1c
Cpd
Cpeb1
Creb3l2
Cryab
Csf1
Csnk1a1
Cul3
Dbnl
Dctn1
Dctn2
Dennd5a
Dgat2
Dicer1
Dixdc1
Dlg1
Dnajc6
Dnm2
Dnm3
Dock1
Dock6
Dpysl2
Dst
Dync1h1
Dync1i2
Dync1li2
Dynll2
Ehd4
Eif4h
DEG
Elmo1
Eml1
Eml2
Eps15
Exoc5
Exoc6b
Fbxl5
Fcho2
Fgfr2
Fign
Fmr1
Fnbp1
Fth1
Fus
Fxr1
Galnt6
Gcc2
Gnai3
Gnb2
Golga7
Gosr2
Gsk3b
Gsn
Hgs
Hhatl
Hnrnpk
Hook3
Hsp90aa1
Ick
Ift80
Inf2
Inpp5f
Insc
Itga4
Itgb1
Itpr1
Itsn2
Kif13a
Kif13b
Kif16b
Kif1a
Kif1b
Kif21a
Kif3c
Kif6
Klhl12
Ktn1
Ldb3
Ldlrad3
Ldlrap1
DEG
Lgi3
Llgl1
Lmbrd1
Lnpep
Lrp1b
Lzts2
M6pr
Map3k11
Map4k2
Map6
Map6d1
Map7
Map7d1
Mapk8ip1
Mapt
Megf10
Mical3
Micall1
Mobp
Mon2
Mrc2
Mrfap1
Mrvi1
Msn
Mtmr2
Mvp
Myh9
Myo1b
Myo1d
Myo1e
Myo6
Naa15
Napa
Nbas
Nbea
Nckap1l
Ncl
Ncoa3
Nde1
Ndor1
Ndrg1
Nedd4
Nek9
Nmral1
Nxt2
Osbpl7
Pafah1b1
Pak1
Pak2
Pard3
DEG
Pcm1
Pdcd6ip
Pde4b
Pear1
Pex5l
Pfn1
Phldb1
Picalm
Pik3r1
Pkp4
Pla2g16
Pla2g4a
Pld1
Plk3
Ppp1r16b
Prkacb
Prkcz
Psrc1
Pten
Ptgds
Ptges3
Ptk2
Pum2
Rab10
Rab11fip2
Rab14
Rab21
Rab22a
Rab2a
Rab31
Rab39b
Rab40c
Rab6a
Rab6b
Rabgap1l
Rac1
Rala
Rap1a
Rasgrp3
Rdx
Rhoa
Rhobtb3
Rock2
Rtn3
S100b
Sass6
Scamp1
Scamp5
Scarb1
Scrn1
DEG
Scrn3
Selm
Senp6
Sept2
Serbp1
Sgk1
Sh3gl3
Sh3kbp1
Shtn1
Slain2
Slc16a1
Slc8a3
Slk
Snx1
Snx2
Snx27
Snx30
Snx33
Snx5
Snx6
Sort1
Specc1
Srcin1
Srd5a1
Ss18
Stam
Stard9
Stmn1
Stmn4
Stx16
Stx4a
Stx6
Stx7
Stxbp2
Stxbp3
Stxbp6
Syde1
Synj1
Synj2
Sys1
Syt11
Syt9
Sytl2
Tbc1d10b
Tbc1d15
Tbc1d16
Tbcd
Tgfa
Tgoln1
Tmed10
DEG
Tnks
Tnks2
Tpd52
Tppp
Tppp3
Tpr
Tra2b
Traf6
Trappc10
Trf
Tsg101
Ttll5
Tuba1a
Tubb4a
Twf1
Tyro3
Uaca
Ube2b
Ube3a
Usp6nl
Vamp3
Vapa
Vbp1
Vldlr
Vps26a
Vps35
Vps52
Vps8
Vtn
Wasf2
Wipf1
Xiap
Xpo1
Xrcc3
Ywhab
Ywhaz
-1.5
1.5
log2 FC
b
vesicle distance CD63-GFP [µm]
35
30
25
20
15
10
5
0
*
anterograde
retrograde
Ctrl siR
Dnmt1 siR
c
vesicle distance Lamp1-mCherry [µm]
35
30
25
20
15
10
5
0
***
anterograde
retrograde
Ctrl siR
Dnmt1 siR

(**a**) Heat-map of significantly up-regulated genes in the *Pvalb-Cre/tdTomato/Dnmt1* KO compared to WT FAC-sorted cortical interneurons associated to the GO term *microtubule-based process*, *vesicle-mediated transport* and *perinuclear region of cytoplasm*. Expression level determined by RNA sequencing ($P < 0.05$, Benjamini-adjusted, n = 9 WT and n = 12 KO mice). (**b**) Boxplot showing the distances covered by antero- and retrograde moving CD63-GFP-positive particles of N2a cells treated with either Ctrl siRNA (n = 14 cells, 34 vesicles) or *Dnmt1* siRNA (n= 14 cells, 38 vesicles) and monitored during the endo-lysosomal vesicle tracking. (Two-sided Student's t-test, * P < 0.05). (**c**) Boxplot showing the distances covered by antero- and retrograde moving LAMP1-mCherry-positive particles of N2a cells treated with either Ctrl siRNA (n = 20 cells, 38 vesicles) or *Dnmt1* siRNA (n= 17 cells, 37 vesicles) and monitored during the endo-lysosomal vesicle tracking. (Two-sided Student's t-test, *** P < 0.001).
DEG = differentially expressed genes, FC = foldchange, siR = siRNA.

Figure S2

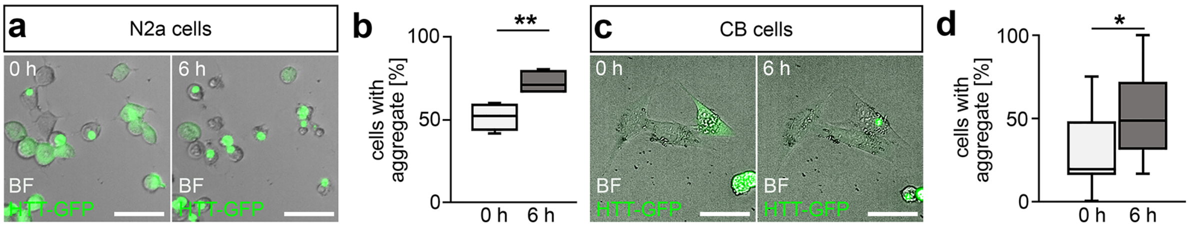

(**a**) Representative microphotographs of control N2a cells expressing GFP-labeled mutant HTT (green) showing the increase of cells with aggregates from timepoint 0 h to 6 h. (**b**) Quantification of cell numbers shown in (**a**) (N = 4 experiments, n = 123 cells; Two-sided Student's t-test, ** P < 0.01). (**c**) Representative microphotographs of control CB cells expressing GFP-labeled mutant HTT (green) showing the increase of cells with aggregates from timepoint 0 h to 6 h. (**d**) Quantification of cell numbers shown in (**c**) (n = 113 cells; Two-sided Student's t-test, * P < 0.05).
N2a = neuroblastoma cells, CB = cerebellar granule cells, BF = bright field, HTT = Huntingtin. Scale bars: 50 µm in (a, c).

Supplementary Movies

Movie 1 – M1_N2a CD63-GFP Ctrl-siR.avi

Example movie of the tracking of CD63-GFP-labeled vesicles during live cell imaging in N2a cells transfected with *CD63-GFP* plasmid and Ctrl siRNA. The arrow is indicating the analyzed transport direction. Scale bar: 10 μm.

Movie 2 – M2_N2a CD63-GFP Dnmt1-siR.avi
Example movie of the tracking of CD63-GFP-labeled vesicles during live cell imaging in N2a cells transfected with *CD63-GFP* plasmid and *Dnmt1* siRNA. The arrow is indicating the analyzed transport direction. Scale bar: 10 μm.

Movie 3 – M3_N2a Lamp1-mCherry Ctrl-siR.avi
Example movie of the tracking of LAMP1-mCherry-positive vesicles during live cell imaging in N2a cells transfected with *Lamp1-mCherry* plasmid and Ctrl siRNA *Dnmt1* siRNA. The arrow is indicating the analyzed transport direction. Scale bar: 10 μm.

Movie 4 – M4_N2a Lamp1-mCherry Dnmt1-siR.avi
Example movie of the tracking of LAMP1-mCherry-positive vesicles during live cell imaging in N2a cells transfected with *Lamp1-mCherry* plasmid and *Dnmt1* siRNA. The arrow is indicating the analyzed transport direction. Scale bar: 10 μm.

Movie 5 – M5_N2a survival 6 h.avi
Example movie of N2a cells expressing GFP-labeled mutant HTT (green) showing higher survivability in cells with aggregate formation (white arrows) than in cells without aggregates (black arrows). Scale bar: 50 μm.

Movie 6 – M6_CB cells without aggregates survival 6 h.avi
Example movie of a CB cell expressing GFP-labeled mutant HTT (green) showing the survivability with aggregate formation. Scale bar: 50 μm.

Movie 7 – M7_CB cells with aggregate formation survival 6 h.avi
Example movie of a CB cell expressing GFP-labeled mutant HTT (green) showing the survivability without aggregates. Scale bar: 50 μm.

Movie 8 – M8_N2a Ctrl-siR Survival 12 h.avi

Example movie of N2a cells treated with Ctrl siRNA expressing GFP-labeled mutant HTT (green) and showing their survival over a time course of 12 h in the HTT-cytotoxicity assay. Scale bar: 50 μm.

Movie 9 – M9_N2a Dnmt1-siR Survival 12 h.avi
Example movie of N2a cells treated with *Dnmt1* siRNA expressing GFP-labeled mutant HTT (green) and showing their survival over a time course of 12 h in the HTT-cytotoxicity assay. Scale bar: 50 μm.

Movie 10 – M10_CB cells Ctrl-siR Survival 12 h.avi
Example movie of CB cells treated with Ctrl siRNA expressing GFP-labeled mutant HTT (green) and showing their survival over a time course of 12 h in the HTT-cytotoxicity assay. Scale bar: 50 μm.

Movie 11 – M11_CB cells Dnmt1-siR Survival 12 h.avi
Example movie of CB cells treated with *Dnmt1* siRNA expressing GFP-labeled mutant HTT (green) and showing their survival over a time course of 12 h in the HTT-cytotoxicity assay. Scale bar: 50 μm.
